# Attempted Abortion by American Hellebore

**Published:** 1858-01

**Authors:** Chas. Palmer

**Affiliations:** Strafford, N. H.


*Attempted abortion by American Hellebore*:—*Messrs. Editors.*—Having noticed a case of attempted abortion by the use of veratrum viride, reported in a late number of the Journal, I extract a similar case from my note-book, which may be of interest in connection with the other.

I was sent for in great haste, and on arriving at the house, found a young woman vomiting freely and complaining of intense pain in the stomach. She was cold and pale. Pulse 40. I inquired what she had taken, and was told "skunk-cabbage." Doubting this, I made further inquiries, and learned that she had taken a teacupful of infusion, prepared from the fresh root of what they thought was skunk-cabbage, (*symplocarpus fœtida*,) but which I found was veratrum viride, gathered by mistake. I was shown what they called a "doctor-book," (a small, popular treatise on medicine, &c.,) in which it was stated that skunk-cabbage would relieve the womb of its contents. The person gathering the root was ignorant of the distinction between the two plants, and hence the mistake. She vomited freely for some time. After the stomach was relieved of its contents, I prescribed an opiate, and she soon recovered. It had no effect on the fœtus in the womb, and in about six months she was delivered of a healthy child.—*Boston Medical Journal.* CHAS. PALMER, M. D.

*Strafford, N. H., Dec.* 15, 1857.

